# Ribitoborate Synergism with Histone Deacetylase Inhibitor Romidepsin as a Potential Treatment for Breast Cancer

**DOI:** 10.3390/ijms27188033

**Published:** 2026-09-09

**Authors:** Ravi Doddapaneni, Jason D. Tucker, Jian Zhang, Pei J. Lu, Qi L. Lu

**Affiliations:** 1 McColl-Lockwood Laboratory for Muscular Dystrophy Research, Atrium Health Musculoskeletal Institute, Carolinas Medical Center, Wake Forest University School of Medicine, Charlotte, NC 28203, USA; 2 Fitzsimons Bioscience Park Center, Z Biotech, LLC, Aurora, CO 80045, USA

**Keywords:** histone deacetylase inhibitors, triple-negative breast cancer, romidepsin, ribitoborate

## Abstract

Triple-negative breast cancer (TNBC) is a subtype associated with poor prognosis and low survival rates, largely due to limited treatment options. In recent years, histone deacetylase (HDAC) inhibitors have emerged as promising anti-cancer candidates due to their attractive epigenetic properties and distinct mechanisms of action but have shown limited success in solid tumors. Here, we explore ribitoborate along with HDAC inhibitors for potential use as a treatment for TNBC. Experiments were performed on the TNBC cell line MDA-MB-231. The results of the study revealed that ribitoborate and romidepsin showed synergistic responses leading to significant arrest of cell proliferation (80%) (*p* < 0.01) as well as migration inhibition. These effects were associated with downregulation of c-Myc, survivin, Bcl-2, and cyclin D1, as well as upregulation of p53 and p21 proteins; notably the changes were significantly greater with the combined treatment than with either ribitoborate or romidepsin alone. Romidepsin and ribitoborate combined treatment time-dependently increased cell death of MDA-MB-231 cells with greater efficiency than romidepsin alone. Cell death by apoptosis was supported by the upregulation of H3k9 and H3k27 acetylation with decreasing proliferation. These results suggest the great potential of the drug combination for treatment of TNBC. The underlying molecular mechanisms for synergy could be further explored for potential development of new combined therapy for breast cancer.

## 1. Introduction

Breast cancer remains the most diagnosed cancer among women worldwide [1]. Breast cancer accounts for roughly one-third of all malignancies in women globally, and mortality rate constituting about 15% of the total number of cases diagnosed [2]. Triple-negative breast cancer (TNBC) lacking expression of estrogen receptor (ER), progesterone receptor (PR), and HER2 constitutes approximately 11–20% of breast cancer patients [3]. TNBC is considered one of the most aggressive forms, characterized by high-grade histology, early recurrence, and a higher prevalence in younger women and African American women [4]. For the treatment of localized breast cancer, surgery and radiation are the first choice, whereas more advanced breast cancers require systemic therapies including chemotherapy, hormone therapy, and targeted drug therapy [5,6,7]. However, the elevated toxicity and resistance to monotherapy limit the effectiveness and safety of treatment. There is a need to explore new combination strategies by targeting the underlying molecular pathways for higher efficacy and lower toxicity.

Over the last decades, our increased understanding of cancer epigenetics has led to new therapeutic approaches to improve patient outcomes. Histone deacetylase (HDAC) inhibitors now represent a very promising epigenetic treatment strategy that reverses the increased expression and activity of HDACs, which are critical for the development and progression of many cancers [8,9,10]. Nevertheless, only a handful of HDAC inhibitors (belinostat, panbinostat, romidepsin and vorinostat) and tucidinostat (which was approved in China and Japan) have been approved to treat mainly hematologic malignancies [11,12,13,14]. While effective in hematological cancers, these HDAC inhibitors have shown limited single-agent efficacy in solid tumors [15,16,17]. To overcome these limitations, both preclinical and clinical studies have examined the combination of HDAC inhibitors with other drugs for synergy in breast cancer [18,19,20]. However, limited progress has been made on the selection of the most suitable combination agent.

We previously reported that supplement of ribitol enhances matriglycan expression in some epithelial cell lines, but the treatment does not significantly inhibit growth, nor provide survival benefit to the cells [21]. However, ribitol is able to act as an adjuvant synergizing with some chemotherapeutic drugs in selective cancer cell lines. The mechanism is not clear but likely related to the involvement of ribitol in cellular metabolism of glycolysis, as suggested in our earlier report [22,23]. Improving cancer cell response to chemotherapeutic drugs by modified sugar adjuvant has long been reported. We hypothesize that further modification of ribitol might synergize with chemotherapeutic drugs with enhanced capacity for killing cancer cells. One example of such a strategy is the application of 2-deoxy-glucose (2-DG), which acts as a glucose mimic inhibiting the function of hexokinase and glucose-6-phosphate isomerase and induces cell death. In addition to glycolysis inhibition, other molecular processes are also affected by 2-DG. Attempts have also been made to improve 2-DG’s drug-like properties, and to generate novel 2-DG analogs as promising new anticancer agents. We therefore hypothesize that chemical modification of ribitol could further enhance the anti-cancer effect. The latest research into boron-containing compounds for cancer has grown significantly, particularly after the FDA approval of bortezomib (Velcade), used to treat multiple myeloma [24]. Utilizing potential anti-cancer properties of borate, here we modified ribitol with boron as ribitoborate to explore its anti-cancer potential. In this study, we examined the role of ribitoborate in combination with HDAC inhibitors against breast cancer cells.

For the first time, we investigated the synergistic cytotoxicity of the HDAC inhibitor in combination with ribitoborate in breast cancer cells and explored their anti-cancer potential as well as mechanisms underlying their synergy. Our results revealed that romidepsin was most efficient in cancer cell cytotoxicity compared to all other screened HDAC inhibitors. The combination of romidepsin with ribitoborate synergistically decreases cancer cell migration and enhances growth inhibition and cell death in the TNBC cells. Further study could elucidate the detailed mechanisms of action and potentially leads to the development of better drug combinations for breast cancer. The results also emphasize the potential of metabolites and their derivatives as therapeutic agents, especially when combined with other drugs.

## 2. Results

**Figure 1:**
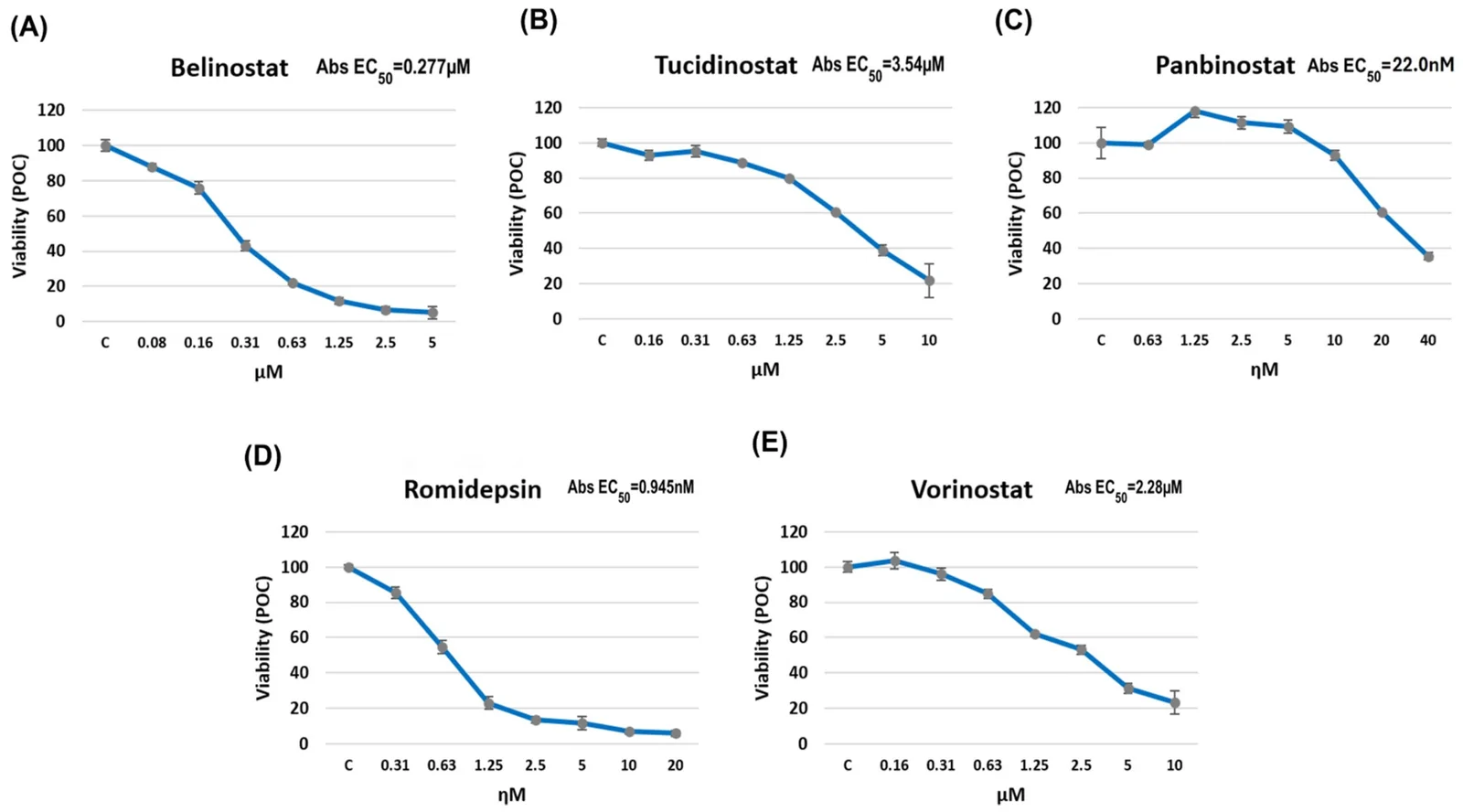
Dose–response curves of MDA-MB-231 breast cancer cells treated with HDAC inhibitors. Cells were seeded in 96-well plates overnight and exposed to a serial concentration of individual drugs for 3 days, as described in methods. Viability of the cells after 72 h treatment with (**A**) belinostat; (**B**) tucidinostat; (**C**) panbinostat; (**D**) romidepsin; (**E**) vorinostat. Rate of cell viability was determined relative to untreated control by ATP CellTiter-Glo assay. Number of replicates = 3.

We first screened the triple-negative breast cancer (TNBC) cell line MDA-MB-231 (from ATCC, Manassas, VA, USA) with FDA-approved (in clinical phases for the treatment of solid tumors) HDAC inhibitors belinostat, panbinostat, romidepsin, vorinostat and tucidinostat. Initial viability assays with serial dilution showed that all HDAC inhibitors reduced the cell viability dose-dependently, ranging from 0.07 µM to 5 µM and from 0.2 µM to 10 µM, achieving EC_50_: 0.27 µM and EC_50_: 2.28 µM for belinostat and vorinostat, respectively (Figure 1). Tucidinostat treatment from the dose range of 0.15 µM to 10 µM decreased the cell viability from 92% (at 0.2 µM) to 21% (at 10 µM) with EC_50_ at 3.54 µM (Figure 1). The MDA-MB-231 cells were most sensitive to the treatment of romidepsin and panbinostat with EC_50_ at 0.945 nM and 22 nM, ranging from 0.3 nM to 20 nM and from 0.6 nM to 40 nM, respectively (Figure 1C,D). Graphs of EC_50_ values of each drug were produced by Combenefit 2.0 software (Figure S1). Therefore, among all the screened HDAC inhibitors, romidepsin was most effective in reducing the viability of MDA-MB-231 cells, achieving 85% killing at 20 nM concentration after 72 h incubation.

### 2.1. Synergistic Studies of HDAC Inhibitors in Combination with Ribitoborate

**Figure 2:**
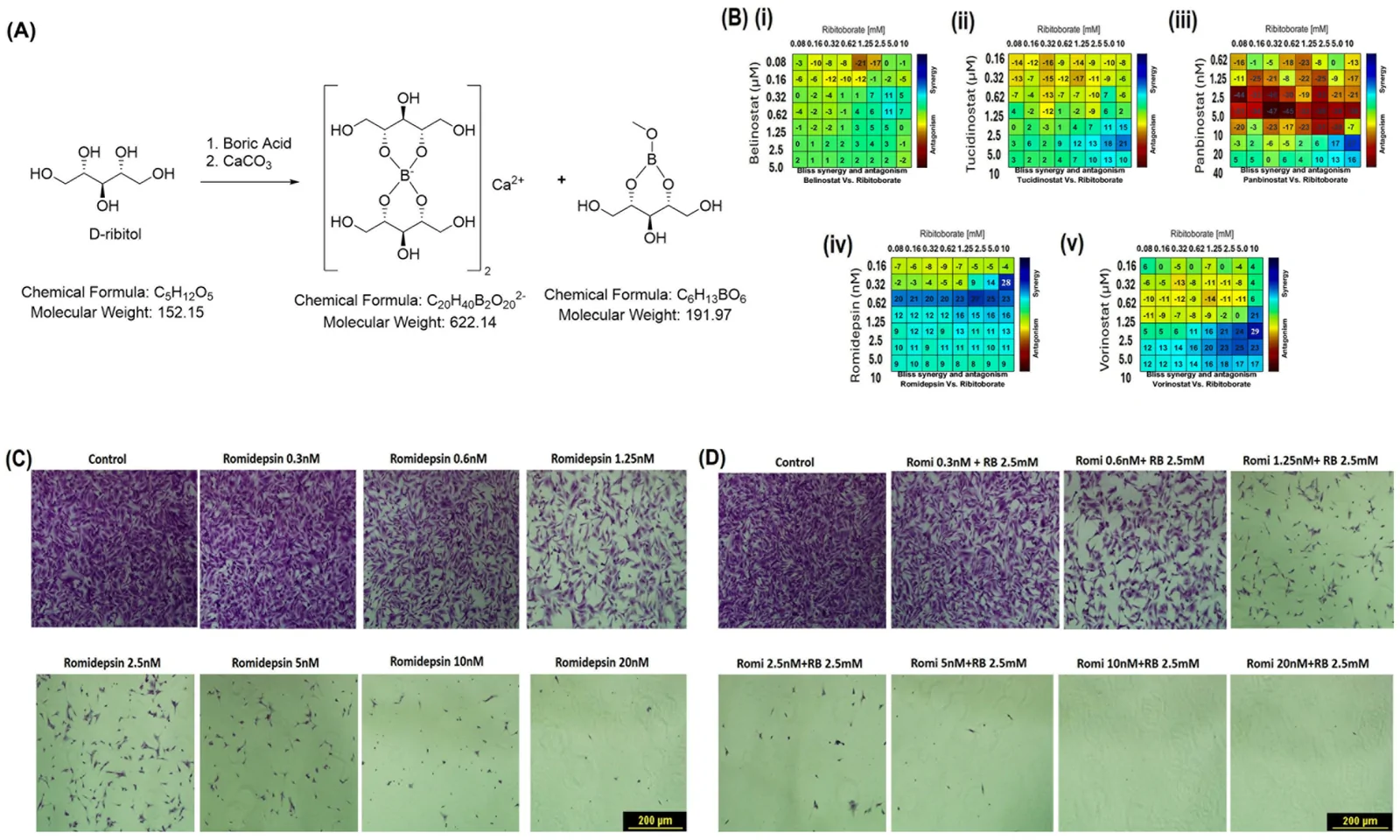
Synergistic effect of HDAC inhibitors (belinostat, romidepsin, tucidinostat, panbinostat and vorinostat) in combination with ribitoborate. Cells were seeded in 96-well plates overnight and exposed to drugs individually, or in combinations. Cell viability was measured using ATP detection (CellTiterGlo assay) after 72 h of incubation. (**A**) Chemical structure and synthesis of ribitoborate; (**B**) synergy matrix plots of cancer cells treated with romidepsin and ribitoborate at a series of concentrations. Blue color indicates synergy between two compounds. Synergy between HDAC inhibitors with ribitoborate was analyzed using Combenefit software (version 2.021). MDA-MB-231 cells were incubated with (**B**) (**i**) belinostat, (**ii**) tucidinostat, (**iii**) panbinostat; (**iv**) romidepsin and (**v**) vorinostat. The graphs were representative of three independent experiments. (**C**) Representative images of breast cancer cells were stained with crystal violet after romidepsin treatment alone for 72 h and (**D**) cells were treated with romidepsin with a wide range of concentrations and constant ribitoborate concentration of 2.5 mM (based on synergy data) combination treatment for 72 h. Images were taken by inverted light microscopy at ×100 magnification (scale bar = 200 µm).

Our previous study demonstrated that ribitol enhances the efficacy of anticancer agents in selected breast cancer cells [22,23]. Here, we synthesized the ribitoborate to investigate its therapeutic efficacy along with HDAC inhibitors. The structure of ribitoborate is shown in Figure 2A and a detailed description of MS/MS and NMR analysis of ribitoborate structure are provided in Supplementary Files as Figure S2. In this study we explored the therapeutic synergy of ribitoborate as an adjuvant in combination with HDAC inhibitors. Ribitoborate alone, up to a concentration of 5 mM, did not exhibit a significant effect on cell viability in MDA-MB-231 cells. To determine the synergy between ribitoborate and HDAC inhibitors described above, we combined a range of concentrations of each HDAC inhibitor with ribitoborate ranging from 0.07 mM to 10 mM. Both drugs were given at the same time for 3 days. Surface matrix plots of cell viability determined by ATP CellTiter-Glo assay were established by Combenefit software. There was no clear synergy when ribitoborate was used in combination with belinostat and tucidinostat at any concentration compared to each HDAC inhibitor alone (Figure 2B(i,ii)). As shown in Figure 2B(iii), an antagonism was observed when ribitoborate (at a wide range of doses) was used in combination with panbinostat, most clearly identified at 2.5 nM and 5 nM. Interestingly, the combination of ribitoborate and romidepsin exhibited enhanced growth inhibition over romidepsin alone in the TNBC cells, as shown in Figure 2B(iv). The ATP CellTiter-Glo assay showed that the combination treatment markedly enhanced romidepsin’s effect starting at very low doses and across the wide dose range of ribitoborate, from 0.07 mM to 10 mM (Figure 2B(iv)). The most significant synergy was detected at 0.625 nM of romidepsin. We further calculated the combination index (CI) to quantify the synergistic interaction between ribitoborate and romidepsin. For all concentrations tested, ribitoborate at 2.5 mM show strong synergy with romidepsin at 0.5 nM concentration (Table S1). Overall, the combination of ribitoborate reduced the EC_50_ dose of romidepsin to 0.5 nM from 0.945 nM when used alone. Synergy was also detected between ribitoborate and vorinostat but at higher concentrations (at 2.5 µM to 5 µM and 10 mM, respectively) of both agents (Figure 2B(v)). As illustrated in Figure 2C,D, the dose-dependent effect of romidepsin and synergy with ribitoborate was also clearly demonstrated with crystal violet staining. These results suggest that ribitoborate could reduce the effective dose of the HDAC inhibitor romidepsin, thus decreasing the potential side effects for treating breast cancer with a reduced dose.

### 2.2. Effect of Romidepsin with Ribitoborate Combination on Cell Proliferation and Migration

**Figure 3:**
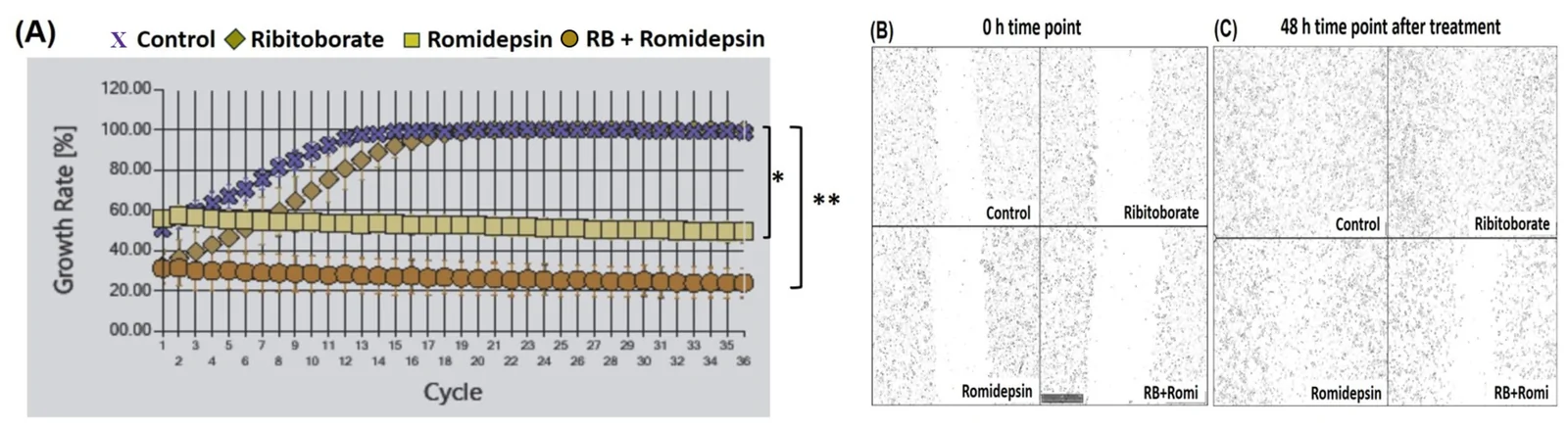
Synergistic effect of ribitoborate and romidepsin on MDA-MB-231 cell proliferation and migration. The treatment of cells divided into 4 groups as control (purple X), ribitoborate (diamond shape), romidepsin (square shape) and the combination of ribitoborate and romidepsin (circle shape) with romidepsin (0.5 nM, half of the EC_50_ concentration) and ribitoborate (2.5 mM) over a period of 72 h. Cells were seeded in 12-well plates overnight and exposed to individual drugs or two-drug combinations and analyzed for cell proliferation and migration by MuviCyte Live-Cell imaging system. (**A**) Growth curve analysis of the cells. (**B**,**C**) Representative images of migration assay before (0 h) and after (72 h) the cells were treated with indicated compounds. Video footage of Live-Cell imaging analysis is attached. Shown are the representative images at 100× magnification (scale bar = 250 µm); untreated cells were used as a control. All data are mean ± SEM. *p* < 0.05 considered significant. * *p* < 0.05; ** *p* < 0.01 compared with untreated control.

To investigate the effect of romidepsin and ribitoborate combination on cell growth, we performed a growth curve analysis under live-cell monitoring (MuviCyte, PerkinElmer, Waltham, MA, USA), which allows precise, automated tracking of proliferation and migration of cells (Figure 3). Ribitoborate (2.5 mM) significantly inhibited growth of MDA-MB-231 cells treated with romidepsin (0.5 nM, half of the EC_50_ concentration) (Figure 3A). A significant reduction (50%) in cell growth was detected with romidepsin treatment alone compared to control and ribitoborate. However, cell growth reduced by 80% (*p* < 0.01) with romidepsin and ribitoborate combination (Figure 3A), a 30% further reduction compared to romidepsin alone (*p* < 0.05). The growth curve also revealed that the synergistic effect occurred soon after the administration of the drugs, and the effect was maintained until the end of the experiment.

We then performed a wound-healing/scratch assay to determine the effect of ribitoborate and in combination with romidepsin (0.5 nM, half of the EC_50_) on migration of the cells. We created cell-free gaps across the center of the wells in a confluent monolayer and monitored the collective movement of cells filling this wound over a period of 72 h with label-free imaging recorded every 2 h (video of automated monitoring of cell migration attached for different treatment groups) (Figure 3B,C). The recorded data generated quantitative changes in wound area, median cell speed and directional persistence. The untreated MDA-MB-231 cells exhibited a complete bridging of the acellular gap over the time, whereas the cells treated with ribitoborate alone slightly delayed the speed of migration but were still able to fill the gap (live recorded video attached). Romidepsin alone showed partial bridging of the gap between the scratch at this concentration. However, cells treated with the combination of ribitoborate and romidepsin show further reduced migration speed after 72 h of treatment when compared to single-agent treatment. These results demonstrate that romidepsin and ribitoborate combination inhibits the proliferation as well as migration of MDA-MB-231 cells in a time-dependent manner, clearly indicating its therapeutic potential.

### 2.3. Effect of Ribitoborate and Romidepsin on Apoptosis

**Figure 4:**
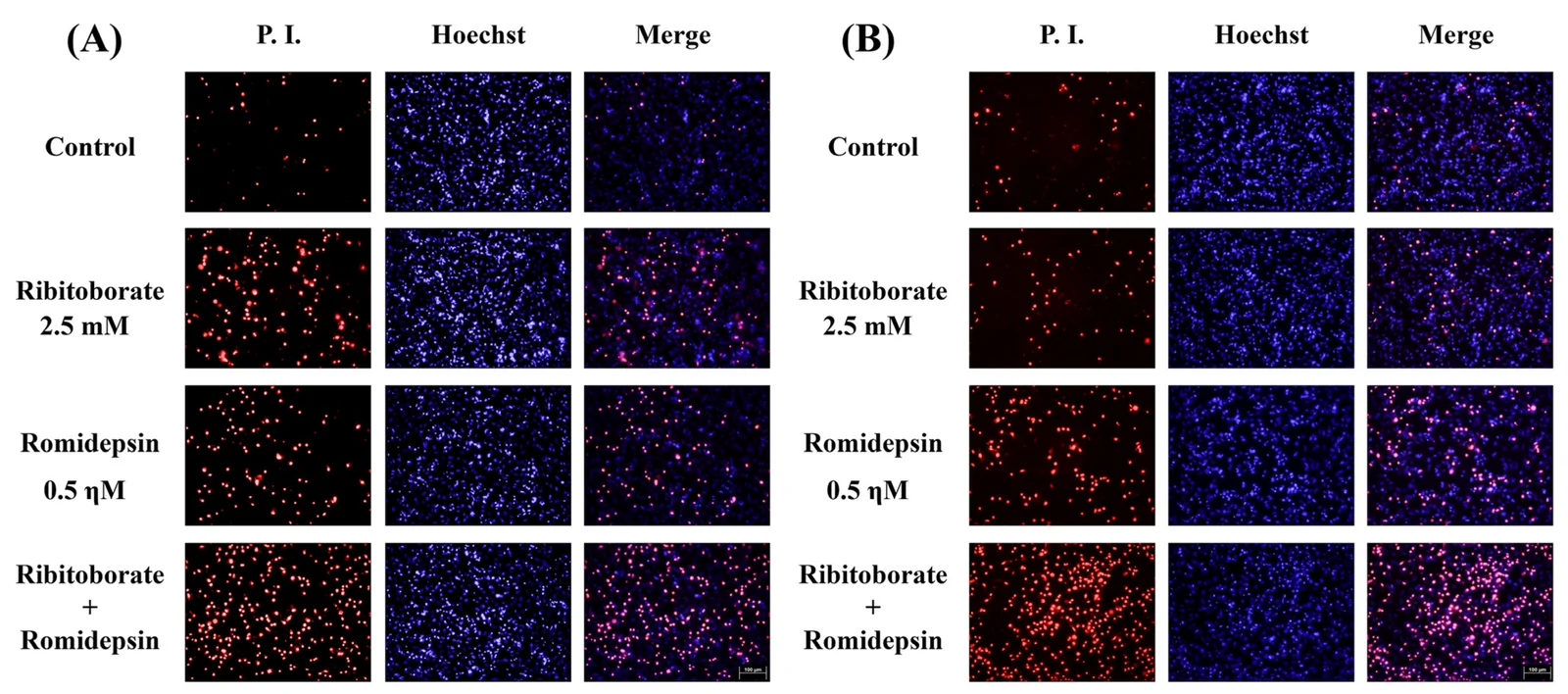
Effects of ribitoborate and romidepsin on apoptosis of MDA-MB-231 cells. Cells were seeded in T75 flasks overnight and exposed to individual drugs or two-drug combinations for apoptosis. Representative images of propidium iodide (PI)/Hoechst 33342 staining of the cells treated with control; ribitoborate 2.5 mM; romidepsin 0.5 nM; romidepsin 0.5 nM and ribitoborate 2.5 mM after (**A**) 48 h and (**B**) 72 h of incubation. Propidium iodide (PI) (red) fluorescence, distinguishing apoptotic cells. Hoechst 33342 (blue) nuclear counterstain, and merged (pink) of PI (red) and Hoechst33342 (blue). Shown are the representative images at 200× magnification (scale bar = 100 µm).

To further explore the mechanisms involved in the drug synergy, we investigated the type of cell death with the drugs at the same doses for cell migration by PI/Hoechst 33342 (Invitrogen, Waltham, MA, USA) staining and examined it by fluorescent microscopy. Representative images of cells stained with Hoechst/PI are shown in Figure 4. Control and ribitoborate-treated cells were uniformly blue in the nucleus (Hoechst-positive and PI-negative cells), indicating they are mostly viable, although a small proportion of cells were stained with PI (red) with condensed or fragmented nuclei, indicating non-viable cells. Romidepsin treatment alone increased the PI-positive cells significantly and the population of cells further increased when used in combination with ribitoborate (Figure 4A,B). The PI-positive cell population increased further from 48 h to 72 h of treatment, especially with the combined treatment (Figure 4B). These results suggest that the combination of ribitoborate and romidepsin induces cell death by apoptosis in a time-dependent manner.

### 2.4. Effect of Romidepsin and Ribitoborate on Survival and Apoptotic Proteins

**Figure 5:**
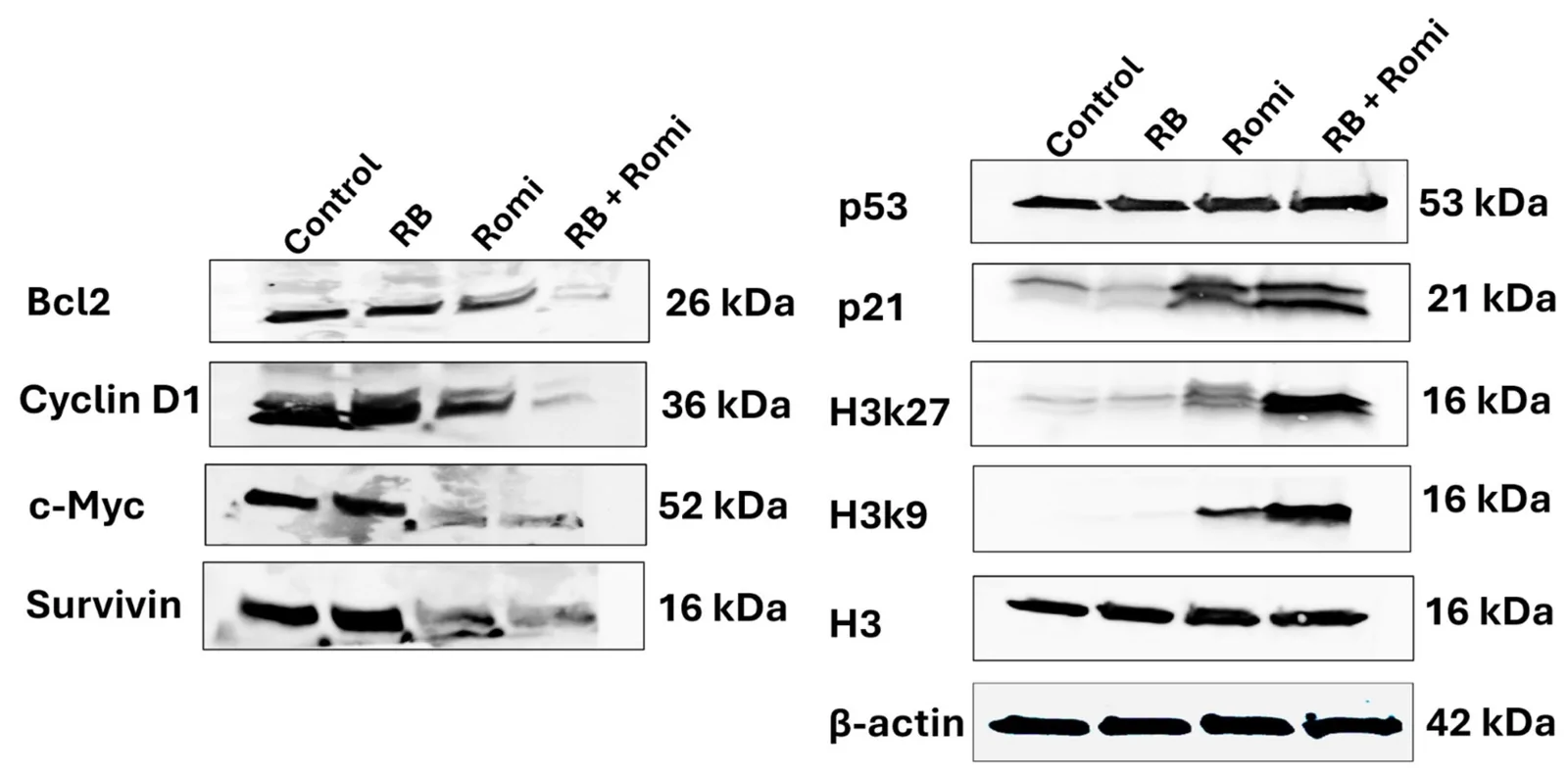
Western blot analysis of drug-mediated effects on various proteins involved in survival and apoptosis of breast cancer cells. Cells were exposed to indicated drug combinations for 72 h and the expression of various proteins was analyzed by Western blot. Cell treatments were as follows: lane 1, control (untreated); lane 2, ribitoborate 2.5 mM; lane 3, romidepsin 0.5 nM; lane 4, romidepsin 0.5 nM and ribitoborate 2.5 mM. Number of replicates = 3.

To investigate the pathways of the observed synergy, we further investigated several proteins known to play a crucial role in apoptosis between the treatment groups. We measured protein expression levels of p53, c-Myc, Bcl-2, survivin, p21 and cyclin D1 (Figure 5). Each protein intensity of signals was quantified by densitometric analysis (Figure S3). The results revealed that single-agent ribitoborate and romidepsin treatment upregulated the expression of the apoptosis master regulator p53 gene slightly and downregulated the expression of the anti-apoptotic protein c-Myc. In particular, the expression of p53 was upregulated significantly (*p* < 0.05) and c-Myc was downregulated significantly (*p* < 0.01) in combination treatment compared to romidepsin alone (Figure 5 and Figure S3). The expression of the anti-apoptotic protein Bcl-2 was lower in romidepsin treatment alone, but further downregulated significantly (*p* < 0.001) in the combination treatment group (Figure 5), consistent with more effective apoptosis with combination than the single agent alone. Survivin, one of the important proteins essential for the survival of cancer cells, was downregulated significantly (*p* < 0.05) in combination compared to single agent. Cyclin D1, a crucial regulator of the cell cycle, was downregulated significantly (*p* < 0.001) in combination compared to control. Another protein, p21, functioning as a tumor suppressor and downstream of p53 activation, was upregulated significantly (*p* < 0.001) in combination compared to single-drug treatment.

Romidepsin alone and combined with ribitoborate also significantly affect the acetylation of histone proteins (Figure 5 and Figure S3). The H3 protein acetylation status of lysine residues 9 and 27 (H3k9 and H3k27) constitutes important switches of many gene expressions. Western blot analysis showed that the H3k9 (*p* < 0.05) and H3k27 (*p* < 0.01) proteins were upregulated significantly in combination treatment compared to control groups. Importantly, the two proteins were both significantly more abundant in the cells treated with both drugs compared to romidepsin alone. These results support the conclusion that the combined ribitoborate and romidepsin treatment arrests cell proliferation and induces apoptosis.

### 2.5. Effect of Romidepsin and Ribitoborate on Cytochrome C Expression

**Figure 6:**
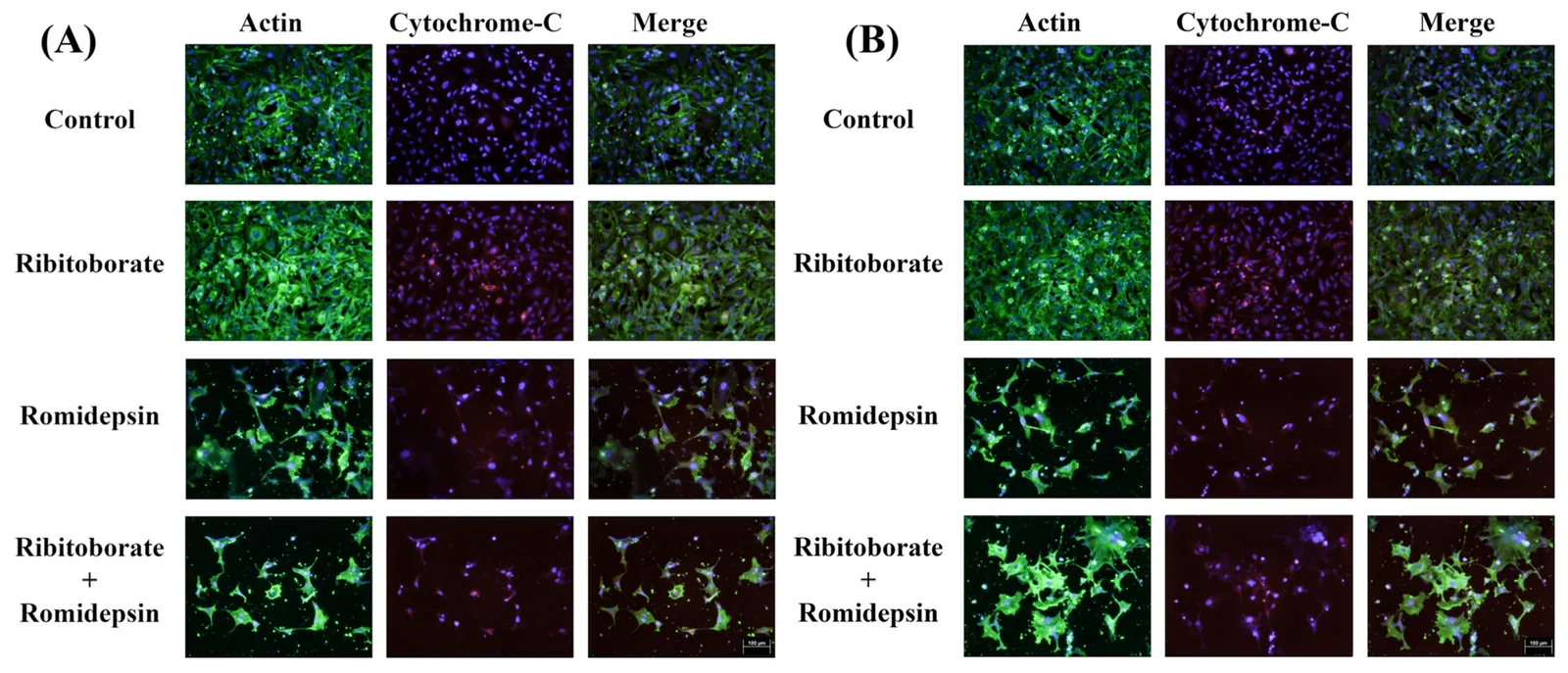
Immunocytochemical analysis of cytochrome C staining after romidepsin and ribitoborate treatment of MDA-MB-231 cells. Immunofluorescence analysis of cytochrome C staining of control, ribitoborate, romidepsin, and combination of ribitoborate and romidepsin. Representative images of MDA-MB-231 cells stained with anti-cytochrome C antibody and photographed at (**A**) 48 h (**B**) 72 h under fluorescent microscopy. Original magnification: 200× (scale bar = 100 µm).

Cytochrome C (Cyt C) is a crucial marker for monitoring apoptosis. We therefore examined its expression and distribution by immunohistochemistry in the cells treated with romidepsin and combination with ribitoborate. Weak cytoplasmic staining for Cyt C expression was observed in a small proportion of the untreated (control) cells (Figure 6). Singal intensity and positive cells with Cyt C expression increased in the ribitoborate-treated cells, but the staining remained largely as diffuse cytoplasmic. Cyt C expression was also detected in the cells treated with romidepsin alone and in combination with ribitoborate. However, the distribution of the staining was different in these 2 treatment groups, with the signal for Cyt C appearing largely within or closely surrounding the nucleus, although weak cytoplasmic staining remains. Similar patterns of expression were observed in the cells treated for 72 h when compared to 48 h (Figure 6A,B). The results of Cyt C moving from the cytoplasmic area into the nucleus are consistent with a role of Cyt C in nuclear condensation, apoptosis and inhibition of cell survival in combination of romidepsin and ribitoborate.

## 3. Discussion

Recent development has shown that targeted treatments, including advanced chemotherapy, antibody–drug conjugates, and metabolic inhibitors can provide therapeutic efficacy against various hematopoietic malignancies [25,26]. However, effective treatment remains few and far between, with limited impact on most solid cancers [27,28]. Currently the best therapeutic outcome can be achieved most likely through combined treatments. Beyond the combination of classic chemotherapy drugs and oncogene inhibitors, a plethora of HDAC inhibitors has been approved for treating hematological tumors [29,30]. In the clinic, however, HDAC inhibitors as single agents have proven less successful for the treatment of solid cancers [31,32,33]. The combination of HDAC inhibitors with other agents could offer a new approach for cancer treatment. Earlier studies have shown that HDAC inhibitors such as trichostatin A, vorinostat and romidepsin can reduce cell viability and induce apoptosis in various types of cancer cells but with considerable side effects in clinics [34]. The synergistic effect of the HDAC inhibitor chidamide and a natural compound such as proanthocyanidin has been reported to inhibit the growth and proliferation of breast cancer cells [35]. Romidepsin in synergy with paclitaxel inhibits metastasis of breast cancer [36]. Synergy allows lower doses of each agent, potentially reducing adverse side effects often seen in high-dose HDAC inhibitor monotherapies [18,37]. This would be especially meaningful if HDAC inhibitors were in combination with less toxic or non-toxic compounds such as metabolites. We previously reported that ribitol as a natural metabolite enhances the efficacy of various chemotherapeutic drugs against breast cancers [23,38]. We now show that ribitoborate, a metabolite derivative with limited cytotoxicity to normal and breast cancer cells when used alone, was able to synergize with romidepsin against breast cancer cells. Overall, our findings suggest that the combination of ribitoborate with romidepsin and possibly other drugs could potentially be a new therapeutic approach to treat TNBC.

Drug synergy is highly selective in partnership and often achieved by screening in selective cell types. Our effort to search for high-potential metabolites as substrates to enhance glycosylation of alpha-dystroglycan for treating specific muscular dystrophy of LGMD2I leads to the synthesis of ribitoborate. Ribitoborate alone shows limited cytotoxicity to normal and cancer cells. Our earlier studies have demonstrated that ribitol is able to enhance drugs in suppressing breast cancer cell survival and migration. We therefore hypothesize that ribitoborate might as well synergize with HDAC inhibitors to suppress growth and enhance cell death of breast cancer selectively. To identify potential drug partners of ribitoborate for synergy, we initially screened several FDA-approved HDAC inhibitors and identified romidepsin as the most potent for breast cancer cells at very low concentration (EC_50_: 0.945 ± 0.021 nM). The combination of ribitoborate with romidepsin decreases EC_50_ by about 50%. This synergy significantly inhibits cell growth and reduces cell migration. Exploring modified metabolites as adjuvant for cancer treatment is advantageous due to their chemical diversity, low toxicity, and availability. While this study is the first time showing the potential of ribitoborate as an adjuvant for breast cancer treatment, further investigation will likely identify more drug partners with synergy for different cancer types.

Romidepsin was discovered more than 30 years ago as a natural product, yet a potent anti-cancer agent [39,40,41]. Its potential as a treatment has been widely tested in many cancers, yet the drug has shown sufficient efficacy only for T cell lymphomas. Only limited efficacy has been reported for solid cancers. The extensive studies exploring its wide applications have nevertheless established its basic mechanism of actions as a histone deacetylase inhibitor [42,43,44]. This action results in widespread histone hyperacetylation, which in turn leads to a cascade event of alteration in gene expression. Accumulating evidence suggests that the most significant consequence relevant to cancer treatment is cell cycle arrest, which is reported to be mediated by activation of p21 gene. The other prominent feature of romidepsin treatment is the enhanced cell death by apoptosis. This effect is linked to the altered expression of several pro- and anti-apoptosis genes. It has been reported that romidepsin upregulates pro-apoptotic proteins Bax and Bak but downregulates anti-apoptotic proteins Bcl-2 and Bcl-xL [45]. These changes can cause the mitochondria to leak cytochrome C into the cytoplasm, thus activating the caspase cascade and cell death. Romidepsin can also increase the production of ROS, causing oxidative stress and further damage to cellular DNA and proteins, enhancing the process of apoptosis. Our results, consistent with the early data, showed that romidepsin, when used alone, increases expression of p21 greatly in the TNBC cells. At the same time, romidepsin decreases the expression of c-Myc, Bcl-2, survivin, cyclin D1. These changes are usually associated with the increased level of ROS, as expected from early study, and may well explain the highly sensitive response of the T-cell receptor (TCR) and T-cell lymphoma (TRBC) to the romidepsin. The increase in levels of H3k9 and H3k27 is also consistent with the fundamental mechanism of romidepsin as an HDAC inhibitor. Interestingly, ribitoborate alone does not change the levels of all the proteins examined clearly. However, when combined with romidepsin, it enhances the effect of romidepsin’s anti-cancer efficacy with almost all proteins examined. Most prominently, ribitoborate further enhances the levels of H3k9, H3k27 and p21 greatly over the levels enhanced by romidepsin alone. Similarly, levels of cyclin D1 and Bcl-2 were further reduced with combined treatment compared to romidepsin alone. Also interesting is that ribitoborate appears to enhance the levels of p53. Our earlier study also reported that ribitol enhances the JQ1 anticancer activity by upregulation of p53 expression and downregulation of anti-apoptotic proteins like c-Myc, Bcl-xL, and Mcl-1 [23]. This would suggest that these expression profiles may be general features of ribitol treatment in a broad range of cells. The role boron plays in the alteration of gene expression and cell death is not clear. In a study of lung cancer cells, treatment with the boron-based drug bortezomib together with romidepsin was reported to contribute to changes in the cell cycle and alter the expression of p21. Further study is clearly required to understand the potential effect of individual components of the new compound.

**Figure 7:**
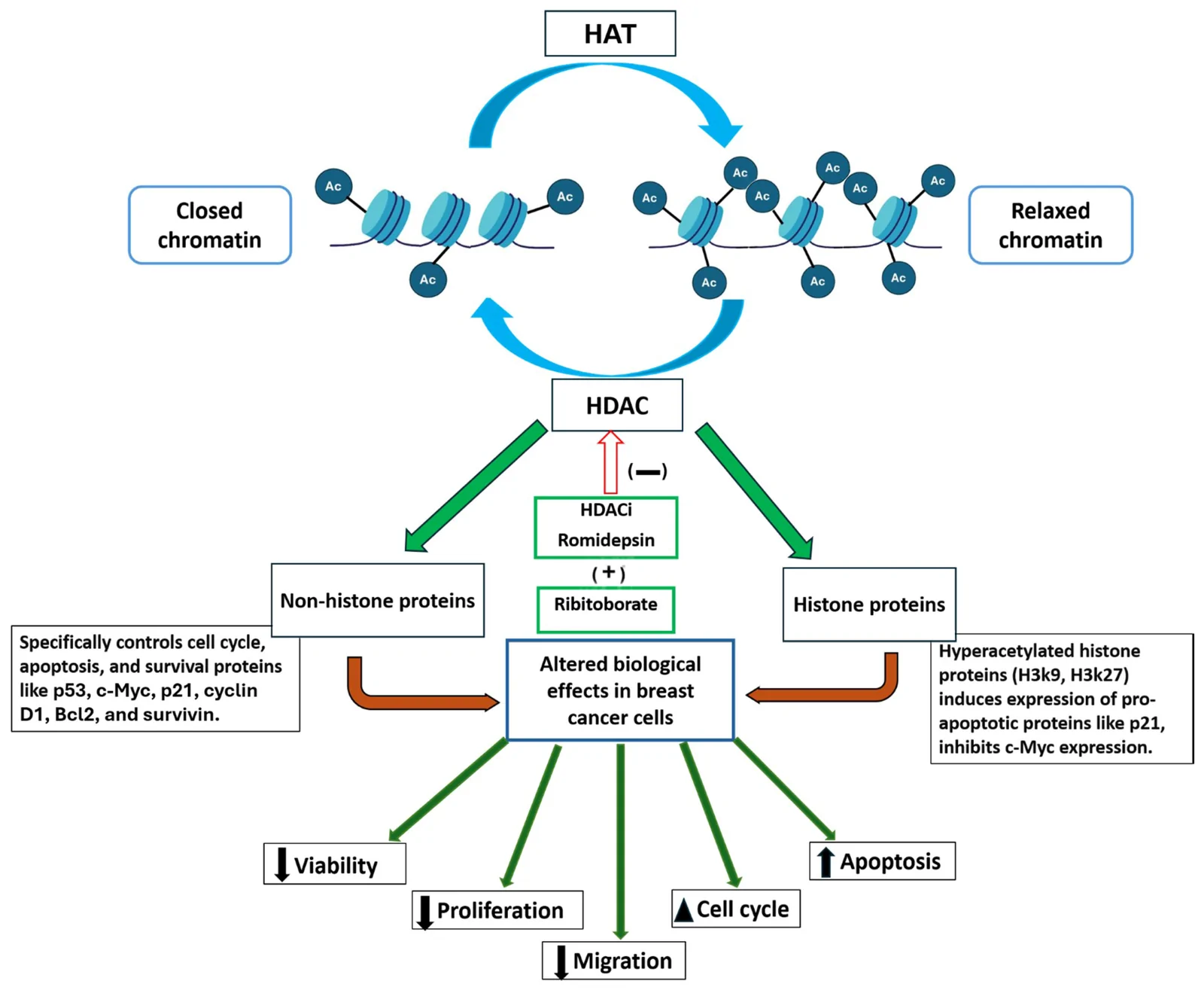
Summary of mechanism of action of romidepsin in combination with ribitoborate on MDA-MB-231 cells. Acetylation of histones alters accessibility of chromatin and allows DNA binding proteins to interact with exposed sites to activate gene transcription and downstream cellular functions. Romidepsin prevents the removal of acetyl groups from histone tails, which results in increased acetylation; this was further enhanced in combination with ribitoborate. This loosens chromatin and generally increases transcription, although they can also repress specific oncogenes. The combination of romidepsin and ribitoborate triggers the various biological effects in cancer cells through modification of histone and non-histone proteins by decreasing viability, inhibiting proliferation, reducing the metastatic potential, arrests the cell cycle and induces the apoptosis of breast cancer cells. Increased ↑; decreased ↓; arrest ▲.

In summary, our results emphasize the potential benefits of ribitoborate as an adjuvant in combination with romidepsin against TNBC (Figure 7). Boron is a rare element with reported benefits to human health. Ribitol is a metabolite in nature and has already been tested both in an animal model and clinical trials for more than 2 years without severe side effects [46,47]. These together suggest that while metabolites may not themselves be singly sufficient as cancer treatment agents, their therapeutic potential as an adjuvant cannot be overemphasized.

## 4. Materials and Methods

### 4.1. Cell Lines, Culture and Ribitoborate Synthesis

The human breast cancer cell line MDA-MB-231 (ATCC-HTB-26) was purchased from ATCC (Manassas, VA, USA). The cells were grown in DMEM-GlutaMAX (Life technologies, Carlsbad, CA, USA) +10% FBS (FBS 10082-147, R&D systems, Minneapolis, MN, USA) at 37 °C in a 10% CO_2_ incubator.

#### Ribitoborate Synthesis

D-ribitol is dissolved in water at room temperature. Boric acid is added to the solution. After it is dissolved with stirring for 30 min, calcium carbonate is added in small portions. After carbon dioxide evolution ceased (20 min), the pH of the reaction mixture was 5~6. A white or light-yellow syrup separates at the bottom of the reaction flask. After keeping the flask in an ice-bath for 1 h, carefully decant the acetone-water solution. The syrup (crude ribitol-boron complex) is treated with acetone/MeOH. Stir the mixture at room temperature for two days to form a suspended white solid. The white solid is filtered off on a Buchner funnel, washed with acetone twice, and air dried. Filter the solution in the Buchner funnel twice and dry the filtrate in a freeze drier. The structure of the ribitoborate was confirmed by nuclear magnetic resonance (NMR) and mass spectrometry (MS/MS). The solid material provided was dissolved (1 mg/mL) in 50:50 MeOH:Water and was introduced via direct infusion (5 µL/min) into a Thermo Eclipse Orbitrap mass spectrometer (Thermofisher Scientific, Waltham, MA, USA). Data was collected at 120,000 resolution in negative mode (−3500 kV; ITT at 325 °C). Further confirmed the ribitoborate structure beyond accurate mass analysis, we fragmented the *m*/*z* 311 and observed the fragments. The expected fragment of *m*/*z* 177 is consistent with a cross-ring fragment of the complex. We also observed further loss of water from this peak, appearing as *m*/*z* 159. Both peaks also appear in the full MS data. NMR analysis of 1H and 13C NMR spectra were recorded on a Bruker 400 MHz NMR spectrometer (Bruker Billerica, Billerica, MA, USA) equipped with a 5-mm PABBO broadband probe. Samples were prepared in D_2_O and analyzed at approximately 26.5 °C. The 1H and 13C spectra were acquired at 400.13 and approximately 100.6 MHz, respectively.

### 4.2. Compound Screening

For primary screening, MDA-MB-231 cells (10,000 cells per well) were seeded in a 96-well plate. We screened a panel of FDA-approved HDAC inhibitors like belinostat, panbinostat, romidepsin, vorinostat and tucidinostat purchased from Selleckchem, Houston, TX, USA. The stocks were further diluted in culture medium at least 500 times (<0.1% DMSO). Cells were treated with single agents with various concentrations. Cell viability was determined after 72 h using CellTiter-Glo reagents (Promega, Madison, WI, USA) following manufacturer’s instructions. Viability was measured with a Biotek Synergy NEO Multi-Label Reader (Agilent, Santa Clara, CA, USA) as a percentage of response relative to cells treated with DMSO alone (0% response).

### 4.3. Cell Viability Assay and Synergy Software

For synergistic studies, MDA-MB-231 cells were treated for 72 h with ribitoborate and combinations with belinostat, panbinostat, romidepsin, vorinostat and tucidinostat in all possible combinations of doses. Cell viability was determined after 72 h using CellTiter-Glo (Promega, Madison, WI, USA) following the manufacturer’s instructions. These results were analyzed using Combenefit software (Version 2.021, Cambridge, UK). We configured a 96-well microplate assay compatible with the Combenefit dual-drug interaction software; cell concentration data, read spectrophotometrically, were submitted to rigorous statistical analysis for synergistic or antagonistic interactions, calculated according to the Bliss synergy model, which is part of the Combenefit package. Combenefit software was used to perform synergism determination. The Combenefit software package calculates and displays synergism–antagonism distributions and computes a variety of metrics from the distributions. The dose–response curve for each of the chemotherapeutic drugs is computed by the software from all biologic replicates of that drug combination. The combination index (CI) was calculated [48] for the analysis of the synergistic, antagonistic or additive effects of the two drugs ribitoborate and romidepsin. The CI was calculated using the formula: CI = [(D)1/(Dx)1] + [(D)2/(Dx)2], in which (D)1 was the concentration of the first drug required to achieve a particular effect in the combination; (Dx)1 was the concentration of the first drug that causes an identical effect alone; (D)2 was the concentration of the second drug that achieves a particular effect in the combination; (Dx)2 was the concentration of the second drug that generates the same effect alone. CI > 1 indicates antagonism, CI = 1 indicates an additive effect and CI < 1 indicates synergy.

Cell proliferation was further evaluated after treatment with ribitoborate and romidepsin by crystal violet assay. Approximately, 100,000 cells were cultured in 6-well plates, incubated for 72 h, and then treated with ribitoborate and romidepsin at concentrations of ½ EC_50_. After 72 h, cells were washed with phosphate-buffered saline (PBS), incubated with the crystal violet solution 0.2% (0.2 g crystal violet powder, Merck, MA, USA) for 5 min, and then rinsed again with deionized water (ddH_2_O). After that, plates were left overnight to dry, and the next day, a phase-contrast microscope was used to take pictures of each well plate.

### 4.4. Proliferation Assay

The antiproliferative activity of ribitoborate and romidepsin was assessed by MuviCyte™ live-cell Imaging System. Breast cancer cells were seeded in 24-well culture plates in complete media and incubated overnight. After overnight incubation, cells were then grouped into control (untreated), ribitoborate (2.5 mM), romidepsin (0.5 nM) and the combination of ribitoborate and romidepsin. Cells were incubated for 72 h in a MuviCyte Live-Cell imaging system (PerkinElmer, Waltham, MA, USA) attached to an incubator at 37 °C in 5% CO_2_. The cell migration was monitored by MuviCyte™ live-cell Imaging System for 72 h. Cell growth plots were captured at 2 h intervals via the MuviCyte Live-Cell imaging software (Version 2.0.26, PerkinElmer, Waltham, MA, USA).

In vitro migration (scratch) assay was carried out in MDA-MB-231 cells. The cells were seeded in 12-well culture plates in complete media and incubated overnight. After reaching confluence, a uniform scratch was made in the center of the well using a micropipette tip, and a baseline image was taken of the entire scratch width. Cells were then treated with half of the EC_50_ concentrations determined for romidepsin. Cells were incubated for 72 h in a MuviCyte Live-Cell imaging system attached to an incubator at 37 °C in 5% CO_2_. Microphotographs were captured at 2 h intervals via the MuviCyte Live-Cell imaging software (Version 2.0.26, PerkinElmer, Waltham, MA, USA) and analyzed.

### 4.5. Co-Staining with Fluorescently Labelled Markers to Detect Apoptosis

The purpose of this experiment was to determine the apoptotic activities of combination therapy in comparison to monotherapy. Briefly, MDA-MB-231 cells were treated for 48 h and 72 h with half of the EC_50_ of romidepsin and combination with ribitoborate. For propidium iodide (PI)/Hoechst 33342 staining, cells plated at 4 × 10^4^ per well after 72 h of culture in experimental conditions were processed by addition of propidium iodide (P3566 Invitrogen, Waltham, MA, USA) at 20 µg/mL and Hoechst 33342 (H3570 Invitrogen, Waltham, MA, USA) at 5 µg/mL. After the addition of dyes, the cells were incubated at room temperature in the dark for 10 min, and fluorescent microscopy images were captured in triplicate or more per treatment on an Olympus BX51/BX52 fluorescence microscope (Opelco, Dulles, VA, USA).

### 4.6. Western Blot

The level of apoptotic proteins was assessed by subjecting 60 µg each of total cell lysates to immunoblot analysis. Cells were lysed in Triton lysis buffer containing 1% Triton X-100, 50 mM Tris pH 8, 150 mM NaCl, 1 mM EDTA, and 1× Protease Inhibitor Cocktail (Sigma-Aldrich, St. Louis, MO, USA). After clarification of the lysates by centrifugation at 13,000 rpm for 10 min at 4 °C, protein concentration of the lysates was measured using the Bradford method (BioRad, Hercules, CA, USA). Samples were then electrophoretically separated on a 4–15% Criterion Tris-HCI 18-well gel (3450028, Bio-Rad Laboratories) and transferred onto a supported nitrocellulose membrane. Immunoblots were probed with primary antibodies c-Myc (SC-40), p53 (SC-126), Santa Cruz Biotechnology, Dallas, TX, USA, and Bcl-2 (AB32370 Abcam, Cambridge, UK), p21 Waf1/Cip1 (12D1) rabbit monoclonal antibody (CST#2947, Cell Signaling, Danvers, MA, USA), survivin (D-8) (SC-17779, Santa Cruz Biotechnology, Dallas, TX, USA) and cyclin D1 (A-12) (SC-8396, Santa Cruz) at 1:1000 dilution in 5% nonfat dry milk/1XTBS-0.05% Tween. Acetyl-Histone H3 (Lys9) (C5B11) rabbit monoclonal antibody #9649 and Acetyl-Histone H3 (Lys27) (D5E4) rabbit monoclonal antibody #8173 both from Cell Signaling, Danvers, MA, USA used in 1:1000 dilution. Rabbit polyclonal antibody to actin (A2066, Sigma-Aldrich, St. Louis, MO, USA) was used at 1:3000 dilution in 5% nonfat dry milk/1XTBS-0.05% Tween as loading control. The blots were incubated with primary antibody overnight at 4 °C. After washing, membranes were subsequently incubated with secondary antibodies of HRP-conjugated goat anti-mouse IgG (1:3000), or goat anti-rabbit IgG (1:3000) in their blocking buffer for 1 h 30 min. Bands were detected using ECL detection Kit NEL 104001EA (PerkinElmer, Waltham, MA, USA) by using chemidoc touch imaging system (ChemiDoc MP, BioRad, Hercules, CA, USA).

### 4.7. Immunocytochemistry (ICC)

Cell cultures for ICC were washed with PBS before fixation with ice-cold methanol for 10 min. Residual methanol was removed by washing with PBS and air-drying. Cells were rehydrated prior to staining procedures with PBS and blocked with 6% bovine serum albumin (BSA) and 2% normal goat serum (NGS) in PBS for 30 min. Primary anti-cytochrome C antibodies (D18C7, Cell Signaling, Danvers, MA, USA) and mouse anti β-actin antibodies (AC-74, Sigma-Aldrich, St. Louis, MO, USA) in 1% BSA at 1:600 dilution were incubated for 4 h at RT or overnight at 4 °C. Samples were washed three times for 10 min with PBS and finally incubated with Alexa Fluor 488-conjugated goat anti-mouse IgG or Alexa Fluor 594-conjugated goat anti-Rabbit IgG secondary antibodies (Life Technologies, Carlsbad, CA, USA) at 1:600 dilution. Samples stained without primary antibody were used as secondary controls.

### 4.8. Statistical Analysis

All data are presented as the mean ± standard error of mean (SEM). The significance of difference in treatment groups was determined using one-way ANOVA with post hoc corrections, where value of *p* < 0.05 between the groups was considered as a statistically significant difference between these groups.

## Data Availability

The original contributions presented in this study are included in the article and Supplementary Materials. Further inquiries can be directed to the corresponding authors.

## Supplementary Materials

The following supporting information can be downloaded at: https://www.mdpi.com/article/10.3390/ijms27188033/s1.
