# Supplementary material for: Ribitoborate Synergism with Histone Deacetylase Inhibitor Romidepsin as a Potential Treatment for Breast Cancer
Source: Int J Mol Sci. 2026 Sep 9;27(18):8033. doi: 10.3390/ijms27188033 (PMC13606832; doi:10.3390/ijms27188033)
Supplement: Supplementary file 1 [file ijms-27-08033-s001.zip › ijms-4461668-supplementary.pdf]

Table S1:

Combination index (CI) values of drug combinations ribitoborate and romidepsin in MDA-MB-231 cells (fractional effect (Fa) 0.6-0.8). Combination index (CI) values for each combination in MDA-MB-231 cells exposed to combination drug treatment. Cells were incubated for 72 hours with the indicated combinations of ribitoborate/romidepsin. CI values less than 1 indicate synergy.

Ribitoborate (mM)	Romidepsin (nM)	Combination Index (CI)
2.5mM	0.5nM	0.51246±0.78
2.5mM	1.0nM	0.82672±0.71
5.0mM	0.5nM	0.71246±0.001
5.0mM	1.0nM	0.91124±0.07

Figure S1:

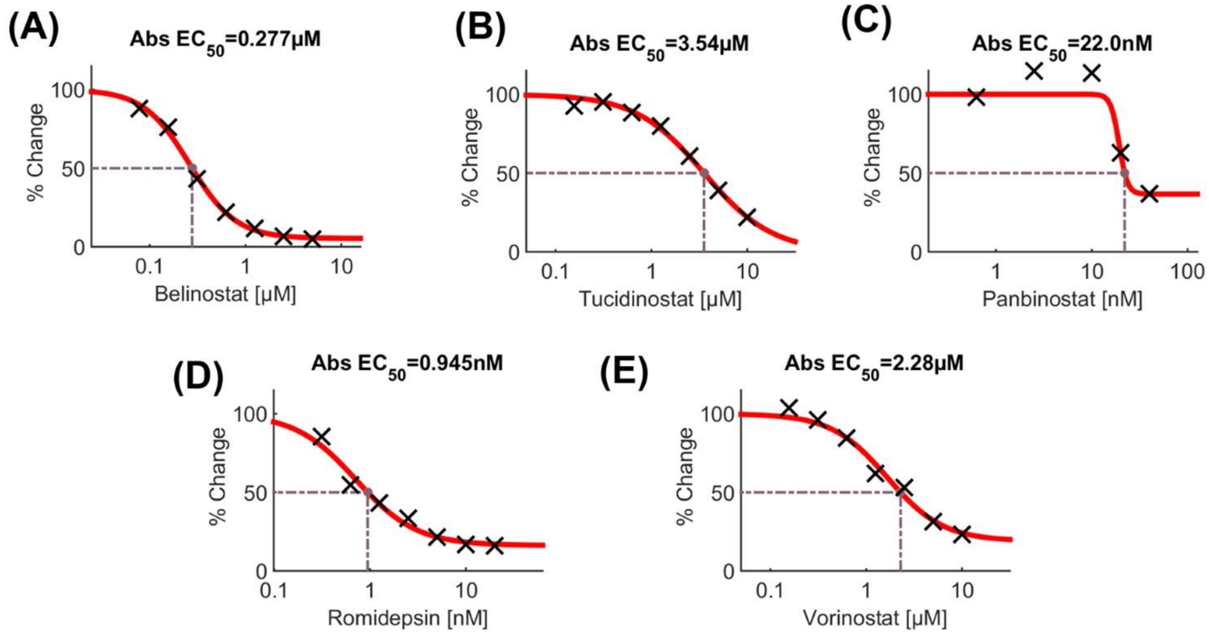

FigureS1: Dose response curves of MDA-MB-231 breast cancer cells treated with HDAC inhibitors. Cells were seeded in 96-well plates overnight and exposed to a serial concentration of individual drugs for 3 days as described in methods. EC_{50} values of (A) Belinostat; B) Tucidinostat; C) Panbinostat; D) Romidepsin; E) Vorinostat were determined using Combenefit 2.0 software. Rate of cell viability was determined relative to untreated control by ATP Cell titer Glo assay. Number of replicates = 3.

Figure S2:

(A)

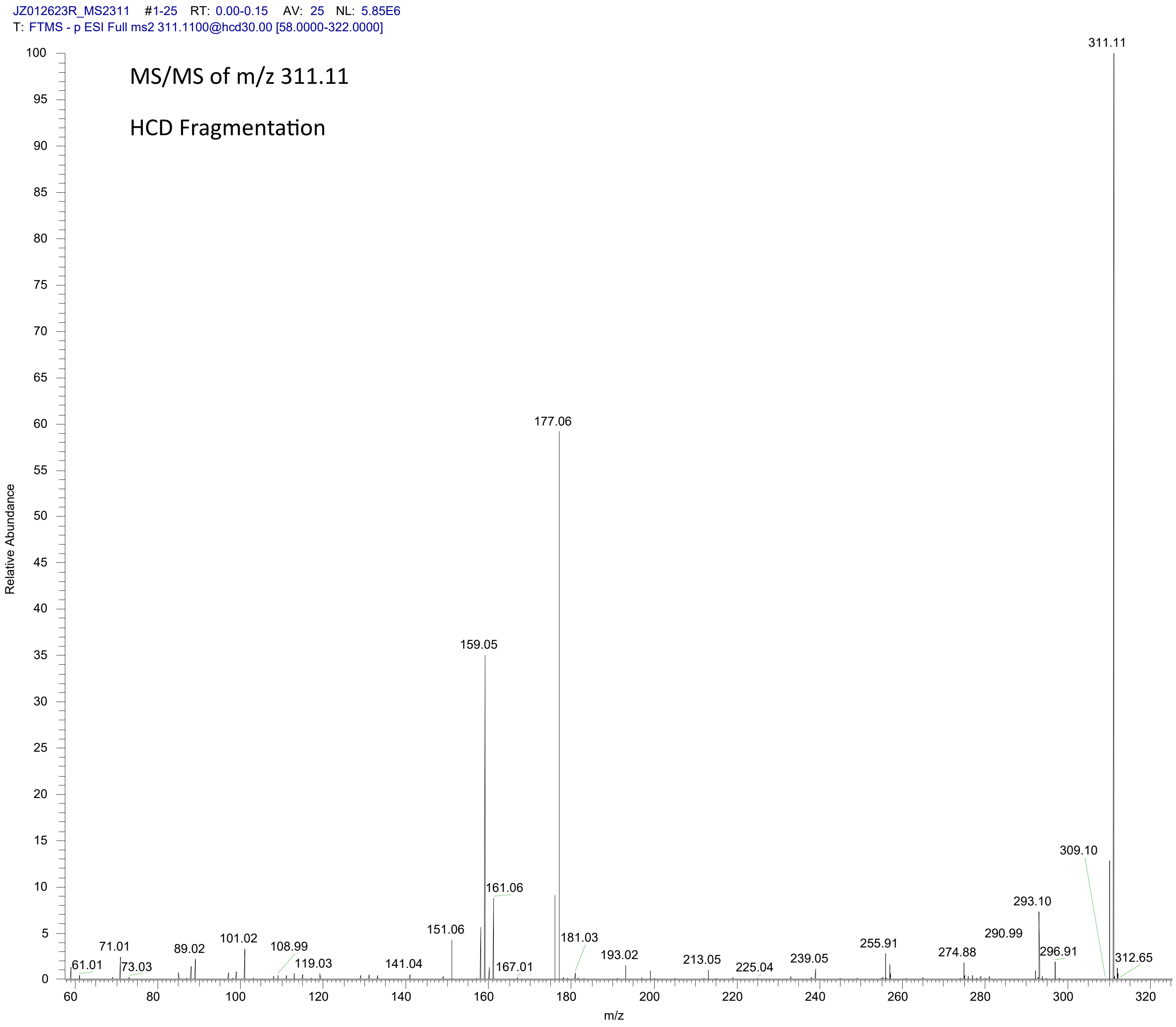
JZ012623R_MS2311 #1-25 RT: 0.00-0.15 AV: 25 NL: 5.85E6
T: FTMS - p ESI Full ms2 311.1100@hcd30.00 [58.0000-322.0000]
MS/MS of m/z 311.11
HCD Fragmentation
Relative Abundance
m/z
311.11
177.06
159.05
309.10
161.06
293.10
151.06
181.03
290.99
71.01
101.02
89.02
108.99
255.91
193.02
274.88
296.91
213.05
239.05
312.65
61.01
73.03
119.03
141.04
167.01
225.04

(B)

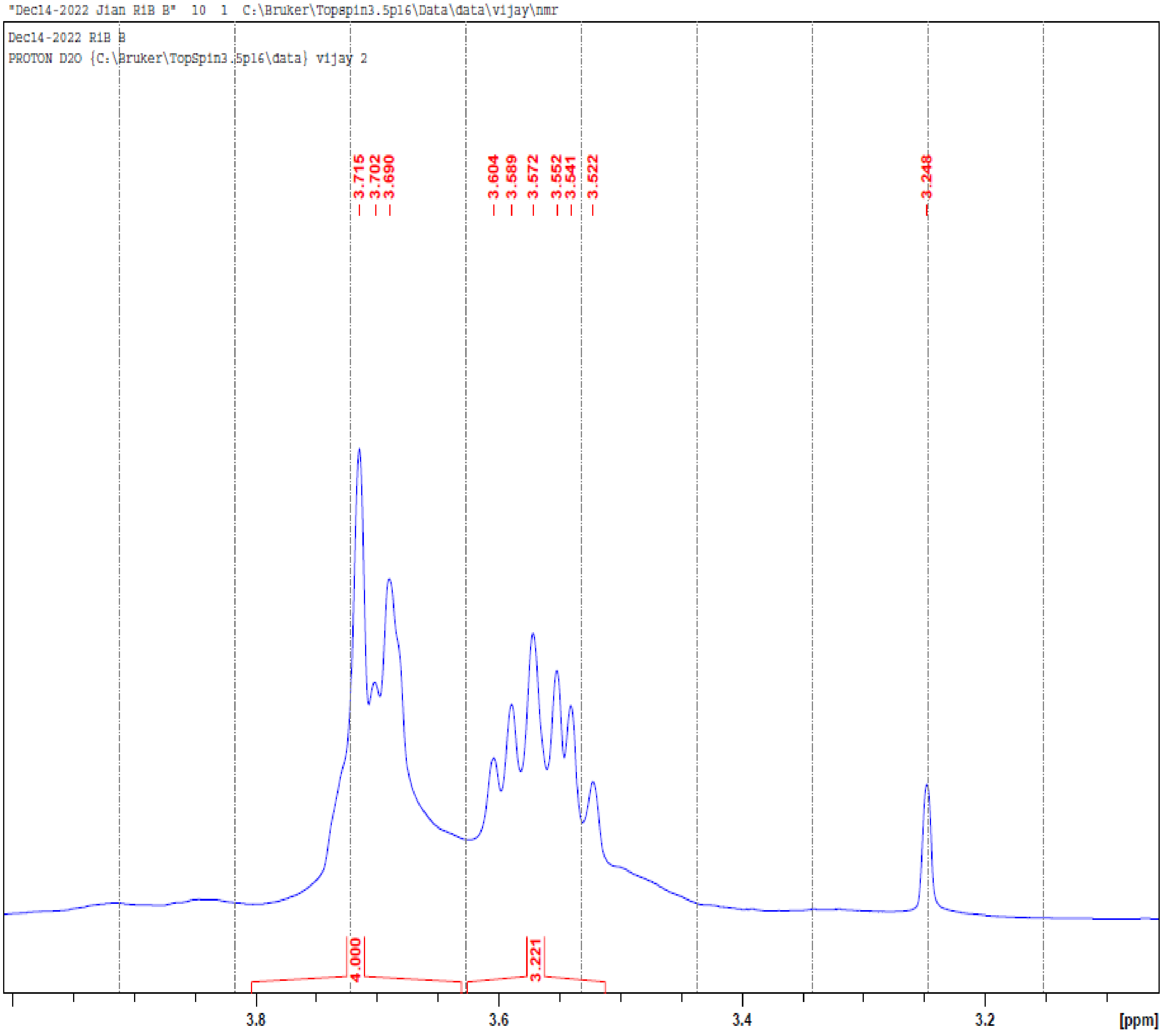
"Dec14-2022 Jian R1B B" 10 1 C:\Bruker\Topspin3.5pl6\Data\data\vijay\nmr
Dec14-2022 R1B B
PROTON D2O {C:\Bruker\TopSpin3.5pl6\data} vijay 2
3.715
3.702
3.690
3.604
3.589
3.572
3.552
3.541
3.522
3.248
4.000
3.221
3.8
3.6
3.4
3.2
[ppm]

(C)

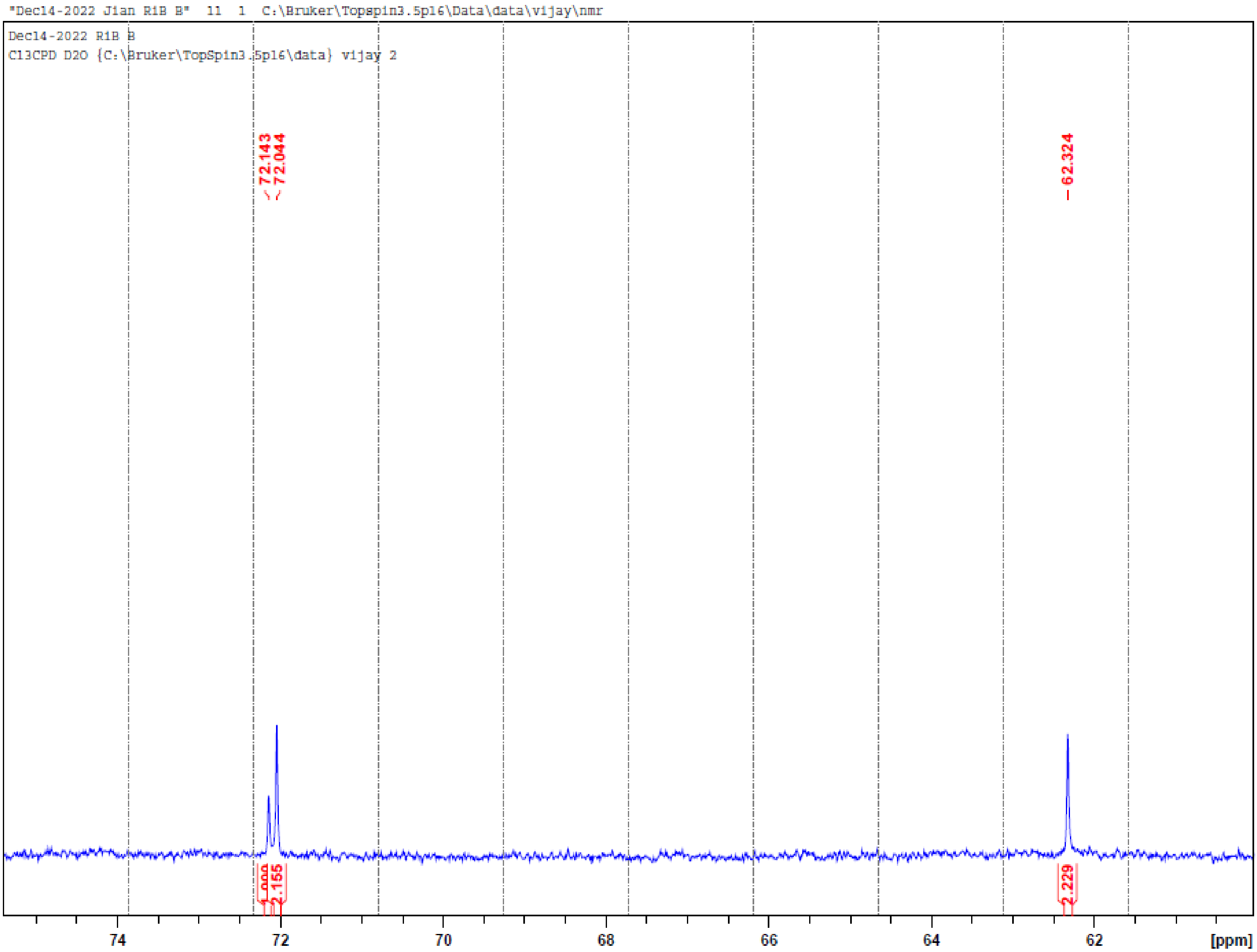

Figure S2: MS/MS and NMR analysis of ribitoborate structure. (A) Confirmed the ribitoborate structure beyond accurate mass analysis, we fragmented the m/z 311 and observed the fragments. The expected fragment of m/z 177 is consistent with a cross-ring fragment of the complex. We also observed further loss of water from this peak, appearing as m/z 159. Both peaks also appear in the full MS data. NMR analysis of (B) 1H and (C) 13C NMR spectra were recorded on a Bruker 400 MHz NMR spectrometer equipped with a 5-mm PABBO broadband probe. Samples were prepared in D_2O and analyzed at approximately 26.5°C. 1H and 13C spectra were acquired at 400.13 and approximately 100.6 MHz, respectively.

Figure S3:

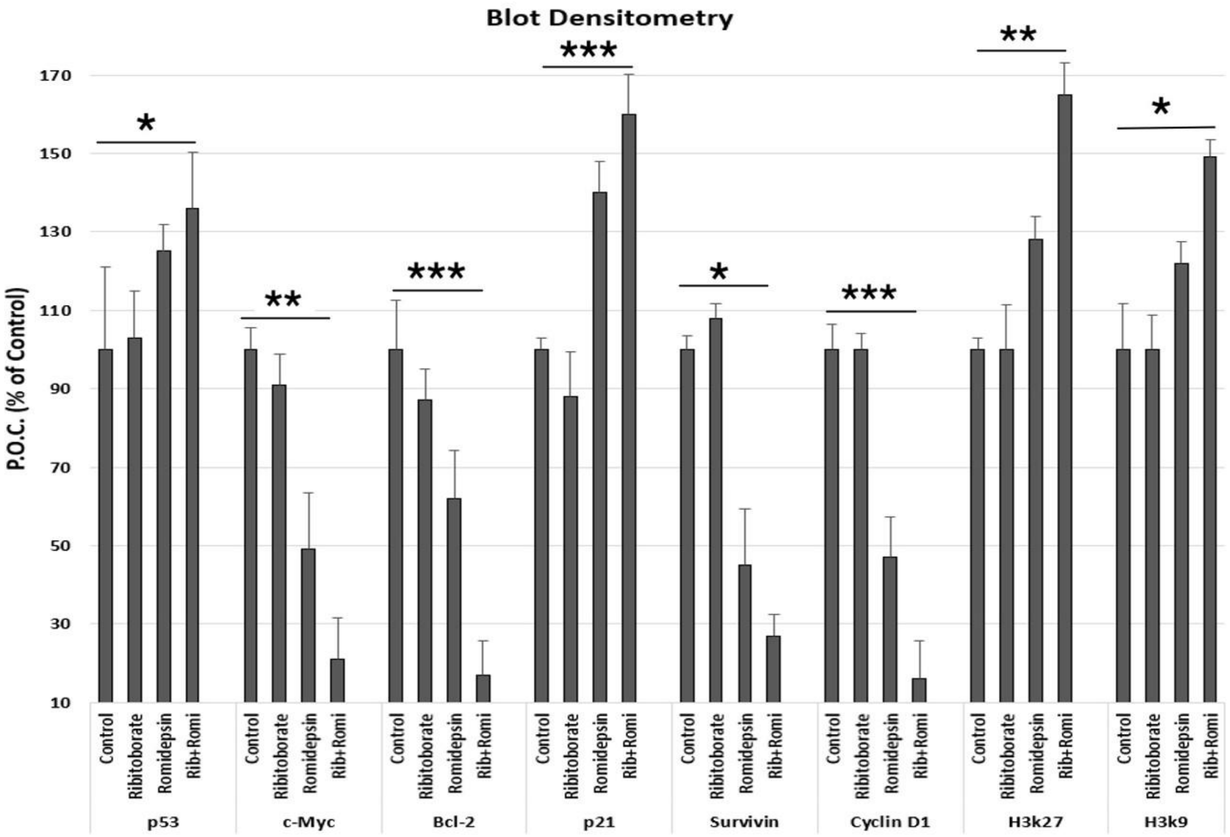

Figure S3: Potential anti-tumor activity of ribitoborate and romidepsin combination on induction of apoptosis in breast cancer cells. Western blot quantification of apoptosis-related proteins in breast cancer cells after treatment. Intensity of signals was quantified by densitometric analysis. Data were calculated from triplicate experiments. Error bars represent mean ± SEM. $p < 0.05$ is considered significant. * $p < 0.05$, ** $p < 0.01$, *** $p < 0.001$ compared with untreated control.
